# Neuroaesthetics: Evolutionary Thinking in Facial Aesthetic Medicine

**DOI:** 10.1111/jocd.70885

**Published:** 2026-05-10

**Authors:** Steven Dayan, Sabrina Fabi

**Affiliations:** ^1^ DeNova Research Chicago Illinois USA; ^2^ Cosmetic Laser Dermatology San Diego California USA

**Keywords:** aesthetic medicine, evolutionary psychology, facial attractiveness, first impressions, mirror neuron system, neuroaesthetics, reward circuitry, skin quality, social perception

## Abstract

**Background:**

Facial aesthetic medicine has traditionally emphasized proportion, symmetry, and structural harmony based on anatomic and cultural ideals. However, rigid adherence to these principles may contribute to overcorrected, unnatural outcomes, challenging perceptions of authenticity. Although patients frequently request to “look natural,” this concept remains poorly defined and is not systematically addressed in training.

**Objective:**

To distinguish beauty, attractiveness, and naturalness and to propose neuroaesthetics as a framework for improving patient‐centered outcomes in aesthetic medicine.

**Methods:**

Narrative synthesis of interdisciplinary literature integrating neuroscience, evolutionary psychology, and aesthetic medicine, with a focus on neural mechanisms underlying attractiveness, reward processing, and social perception.

**Results:**

Perceptions of attractiveness and naturalness are mediated by neural systems involved in reward valuation, rapid first‐impression formation, and embodied simulation. Overreliance on static structural ideals may overlook dynamic cues critical to social and emotional evaluation. Neuroaesthetic principles provide a biologically informed framework for aligning aesthetic interventions with perceptual and psychosocial responses.

**Conclusions:**

Incorporating neuroaesthetics represents a shift from structural optimization toward outcomes that prioritize authenticity, emotional resonance, and social perception. As patient expectations evolve, aesthetic providers should integrate these principles to achieve more natural and meaningful results.

## 1 Introduction

For centuries, aesthetic judgment has been grounded in proportion, symmetry, and harmony. From classical canons of beauty to the neoclassical golden ratio, facial aesthetic medicine has operated within a structural paradigm: anatomic deviations are corrected to meet ideals [1]. This approach is still useful and gives the novice injector or surgeon a scaffold to produce measurable, often impressive results. But it also misleads if pursued dogmatically. Structural refinement alone does not explain why faces are perceived as warm, trustworthy, youthful, or elegant, nor why small changes can disproportionately influence first impressions and social outcomes [2, 3, 4]. It also does not explain why technically “correct” interventions still feel artificial. The face is not simply a collection of proportions; it is a biologically purposed social stimulator tuned for facial identity, affective valuation, and interpersonal prediction. Neuroscience adds an important missing layer to this clinical reality. Functional neuroimaging shows that facial attractiveness engages circuitry involved in reward processing including the medial orbitofrontal, prefrontal cortex and the ventral striatum/nucleus accumbens [5, 6, 7, 8]. In simple terms, beauty is not only seen; it is experienced as value.

To discuss this rigorously we need to define our terms. Beauty and attractiveness are often treated as synonyms, but for academic medicine purposes they need to be separated. Beauty is an evolutionarily preserved signal that suggests vitality, fertility, and genetic fitness and is perceived outside conscious awareness. Attraction is broader. It includes beauty, but also the cognitive dimension of what draws our attention and interest. Attraction is shaped by cultural nuance, adornment, hairstyle, posture, facial movement, and perhaps most importantly, confidence. In essence, you “feel” beauty, but you “think” attraction [9].

This distinction matters because those who focus narrowly on a more “ideal” beauty metric may achieve a more “perfect” face but one that is unattractive and disquieting. Cosmetic patients seek not to be more beautiful but to be more attractive and win the psychosocial benefits that are inclusive of that goal. That means improving physical features without stripping away identity, authenticity, emotional expressiveness, or cultural specificity. Neuroaesthetic science explains the neural processing and perceptual psychology behind the beauty/attractiveness difference. A multi‐dimensional attractiveness goal takes into account the social cognition research revealing that faces trigger rapid judgments of trustworthiness, dominance, competence, and approachability within a hundredth of a second, and before deliberate reasoning begins [10, 11]. The amygdala, superior temporal regions, ventral striatum, orbital and medial prefrontal cortex networks are involved in fast social evaluation and reward centers activation [12]. This neural network comprised of both primitive and evolutionary evolved regions of the brain is recruited. Neuroaesthetic thinking helps clinicians move beyond a purely topographical one‐dimensional structural beauty model toward one that is complex, perceptual, affective, and socially grounded with a goal to deliver a more attractive individual. Neuroaesthetics offers the logical and empirical reasoning why natural outcomes are so strongly preferred, why overcorrection creates unease and why skin quality and movement matter as much as contour. Clinicians, neuroaesthetically guided, are better equipped to identify and treat those most likely to benefit from a cosmetic intervention.

## 2 Neuroaesthetics as a Foundational Framework

Any discussion of neuroaesthetics begins with Semir Zeki's work showing that beauty is not just philosophical but a measurable brain event that is much broader than aesthetic medicine. Across visual art, music, and even mathematics, Zeki and colleagues showed that the experience of beauty correlates with activity in the medial orbitofrontal cortex, a region involved in reward and valuation [13, 14] This is highly relevant to facial aesthetics in which an observer's brain processes the relationships between the eyes, nose, lips, brow, jawline, skin, and how their movement coordinates. It also helps explain why excess, misplaced filler, toxin or exaggerated surgical changes that alter facial movements can make a face feel “off,” even when the individual parts look improved on their own.

Chatterjee and Vartanian later proposed the “Aesthetic Triad,” an aesthetic experience model derived from the interaction of three systems: sensory–motor, which processes what we see and how the face moves; emotion–valuation, which assigns salience and pleasure; and meaning–knowledge, which shapes interpretation through memory, expertise, context, identity, and culture [15]. This model fits facial aesthetic medicine well because our treatments affect all three at once. We change visible form and movement, influence how a face is valued, and alter that which a face communicates. Facial aesthetics cannot be reduced to geometry needing to be corrected; rather, it serves as a messaging tool directed into a perceptual social system that rapidly asks: does this face look healthy, approachable, authentic, rewarding, attractive?

## 3 Evolutionary Pressures Shaping Facial Preference

Under good‐genes and mate‐quality models, facial cues such as symmetry, averageness, skin quality, sexual dimorphism, and age‐linked traits function as indicators of genetic fitness and disease resistance [16, 17]. Faces that appear healthy historically have signaled reproductive and survival advantages. Second, parasite avoidance theories suggest that humans evolved sensitivity to visible cues of biologic compromise. Skin irregularity, textural disruption, discoloration, and asymmetry carry meaningful survival information in ancestral environments. Third, the face is not just a mating signal. It is also a platform for social prediction, supporting kin recognition, threat detection, and status evaluation. Humans likely evolved to use faces not only to answer, “Is this a desirable mate?” but also, “Is this person safe, sick, dominant, familiar, kin or emotionally available?” The modern face is therefore a multidimensional signaling device. And inherent to all humans is an incredibly adept sensory system that reads morphological encoded facial clues beneath comprehension. It is the reason why we can feel “something is off” when we look at someone's face even if we don't know why. When unaware clinicians attempt to improve skin quality or restore tissue loss that is out of sync with age or vitality, they may be changing signals historically tied to health, vigor, reproductive value, and social readability. Unfortunately, it is the reason why many of the universal “paint‐by‐number” approaches of “x” amount of toxin units or “x” milliliters of filler per area may not result in best practices. If treatment introduces biologically implausible contour transitions, fixed expressions, or feature combinations that no longer fit an individual's age, movement, or identity, observers tuned for realism and authenticity may generate unease rather than attraction. A neuroaesthetic‐evolutionary approach to facial aesthetics guides the clinician to avoid this trap.

## 4 Skin Quality, Surface Reflectance, and Neural Activation

Traditional aesthetic analysis emphasizes bone, soft tissue volume, and proportions. Yet a growing body of work suggests that surface quality plays a disproportionately large role in perceived attractiveness. Skin homogeneity, reflectance, texture, and radiance influence attractiveness judgments and first impressions [18]. Sakano and colleagues combined psychophysics and fMRI to examine how skin reflection type, matte, oily‐shiny, or radiant influences attractiveness judgments and brain response. In their study, attractiveness ratings were highest for radiant skin, followed by oily‐shiny and then matte [19]. Related experimental work supports that improving skin homogeneity enhances perceived attractiveness, often without participants consciously identifying what changed [20]. Together, these findings reinforce what experienced clinicians see every day: slight to modest improvements in skin clarity, luminosity, and texture can generate disproportionately positive observer responses. From a neuroaesthetic perspective, homogenous naturally reflective skin enhances perceptual fluency. The clinical translation is simple: faces that are easier for the brain to process tend to be judged more favorably.

Skin embryologically derived from ectoderm similar to the nervous system is embodied with millions of free nerve endings and multiple neuropeptides and neurohormones common to the central nervous system, positioning it as an underappreciated but critical component to how we subconsciously interact with our environment and others. Pilot observational work conducted in 2019 indicated the visually blind's ability to detect beauty raises provocative questions about a yet to be defined extra sensory subconscious ability to interpret the health and vitality of others in close proximity [15]. The data remain preliminary and should be interpreted cautiously. But, it has to be considered an invitation to broaden inquiry into an embodied unrecognized subconscious aesthetic experience linking all humans and a potentially advantageous differentiation from an inevitable world of inanimate, insentient robots.

## 5 Perceptual Fluency, Predictive Coding, and Naturalness

Predictive coding describes how the brain's fusiform face area and associated ventral‐stream systems play a holistic role in the processing of what it perceives by comparing incoming facial signals against learned priors [21]. Faces that conform sufficiently are processed efficiently. Faces that deviate generate conflict. Excessive immobility, age‐incongruent fullness, or a mismatch between static form and dynamic expression may increase the discrepancy between expected and perceived facial states. Naturalness is not simply “less treatment.” it is the preservation of configural coherence. A face may undergo substantial treatment and still read natural if it preserves a believable face. Conversely, relatively modest treatment may look unnatural if it introduces discordance among contour, movement, identity, and age. This may be one reason lay observers often use imprecise but revealing language such as “she looks off,” or “not like herself.” A person may have idealized proportions, symmetry, or flawless skin, but if those features no longer fit the age, culture, or emotional context of the individual, the brain detects conflict and the final perception becomes negative. This explains why subliminal aesthetic changes can produce disproportionately positive outcomes and overcorrection often appears uncanny even when no single feature is grossly abnormal in isolation. It also aligns with published work showing that perceived naturalness strongly influences how patients are judged by observers after treatment [22].

## 6 Trained Eye: Expertise, Pattern Recognition, and Clinical Judgment

Neuroscience supports the notion that art experts process art differently from novices. The experienced attend less to superficial features and more to deeper structural relationships showing more efficient neural processing, different emotional weighting, and greater capacity to extract meaning from abstract visual stimuli [23, 24] Perhaps this same principle applies to the development of the aesthetic injector, plastic surgeon, or aesthetic physician? A trained eye is not simply good taste. It is a form of perceptual expertise built over time through repetition, observation, correction of mistakes, and deeper conceptual training. The expert clinician may see relationships the novice is yet to appreciate: how one change in the midface will alter the perioral region; how slight overfill in the deep medial cheek may change emotional expression; how skin, contour, and movement must read together; and how a technically possible treatment may still be perceptually wrong. Anatomy tells us where we can put product; neuroaesthetic expertise tells us whether we should, what that change means, and how that face will be perceived. Experience and deeper neuroaesthetic training do not just improve technical skills; it also refines visual judgment, pattern recognition, restraint, and strategy. In that sense, neuroaesthetic science gives a theoretical home to what experienced injectors and surgeons often do intuitively.

## 7 Rapid Trait Attribution and First Impressions

The importance becomes even clearer when we consider how quickly the face is judged. Humans form first impressions in less than 100 milliseconds, and those impressions correlated strongly with impressions formed after longer viewing [11]. Such judgments recruit fast, evolutionary ancient systems involved in social evaluation, not only for beauty, but for cues relevant to safety, intention, health, and social value [9, 11]. Subtle less than 1 mm changes in brow position, periorbital contour, lip posture, jawline definition, and skin quality can shift impressions of authority, warmth, fatigue, competence, or threat. Most often these inferences occur outside conscious awareness [10]. Structurally, a tired face may be read as sad or disengaged and a tense brow as angry before a word is spoken, explaining why cosmetic interventions can influence how faces are socially perceived [3]. Aesthetic procedures do not simply make patients “look better,” they recalibrate social predictions that occur instantly.

## 8 Embodied Simulation, Emotional Resonance, and Movement

Social connection is highly dependent on emotional resonance and embodied simulation. Described by Gallese and colleagues, observers mimic and internalize the expressions and emotions of those with whom they interact through their own sensory–motor and visceromotor systems [25]. Subtle facial mimicry supports emotional contagion and empathic access [26, 27]. Treatments that preserve congruent movement and authentic expression help maintain emotional signaling integrity. Conversely, rigidity or an unnatural transition between rest and motion may disrupt the observer's embodied access to the face and cause a social disconnect. The face no longer behaves in a way the observer can easily simulate. It becomes emotionally less readable. Etcoff and colleagues offer a relevant caution. In a study of botulinum toxin treatment to the lateral orbicularis oculi, smiles were judged as less genuinely felt [28]. The implication is not that neuromodulators are harmful to social perception; rather, total immobilization of regions critical to an authentic “Duchenne smile” may weaken cues of a genuine positive affect. This argues for moderation when treating lateral canthal lines of the face with botulinum toxin. Additionally, published observer reporter outcome (ORO's) data supports that only outcomes judged as “natural” after filler treatment were associated with positive attractiveness [22].

## 9 Facial Feedback, Interoception, and the Treated Self

The treated face also influences the patient's affect through their internal facial‐feedback mechanism. Expressions precede emotions through somatic and interoceptive signaling. In aesthetic practice, if we reduce hyperactive negative expressions we may reduce self‐perceived fatigue, distress, or anger. Most clinicians can recount patients reporting they not only look less tired but feel less burdened or happier following a botulinum toxin treatment to the glabellar area. This is not simply anecdotal; a wealth of evidence supports that botulinum toxin treatment may reduce depressive symptoms by inhibiting the corrugators from frowning and corroborating evidence supports an improvement in self‐esteem in those treated with botulinum toxins as well [29]. Transferring this thinking to smile, it has been demonstrated that placing hyaluronic filler to elevate the corners of the mouth can improve first impressions while also being associated with mood elevation [30]. It may be that by inhibiting a frown, or mechanically encouraging a smile with soft tissue fillers alters not only appearance, but also an individual's emotional experience.

Additional support, albeit more indirectly, for the broader concept that facial intervention may influence mood comes from the vascular theory of emotion proposed by Zajonc and colleagues. They argued that facial muscular activity can alter venous return and brain temperature through effects on the cavernous sinus, thereby influencing affective state [31]. This finding becomes especially relevant to aesthetics, overfilling the deep medial cheek fat pad, resulting in an unappealing unnatural “apple cheek” syndrome [32], that may also reduce facial venous drainage influencing brain temperature and creating mood shift. It is not unreasonable to ask whether aggressive deep medial cheek filling might affect more than appearance alone. At minimum, this kind of reasoning shows how a neuroaesthetic lens pushes us to think more carefully about where we inject, how much we use, and what broader consequences may follow.

## 10 Peak Shift and the Optimization of Facial Signals

Another principle from neuroaesthetics that helps explain both the success and failure of aesthetic intervention is the peak‐shift effect well described in the art literature [33]. Peak shift refers to the tendency of perceptual systems to respond more strongly to an exaggerated version of a preferred stimulus than to the original stimulus itself. In facial aesthetics, this suggests that humans may prefer faces that slightly amplify key signals associated with beauty, fertility, and sexual dimorphism. This provides an explanation why modest aesthetic amplification of the lips, breasts, or buttocks can produce disproportionately positive perceptual effects. But the same principle predicts its own limitation. When exaggeration exceeds a biologically plausible range, it may disrupt configural processing, violate perceptual expectations, and trigger negative appraisal. The most successful outcomes occur not at the extremes, but at the point where signal enhancement remains within the bounds of credibility. But this concept proves challenging to teach when there is a temptation by both the patient and provider to push the boundaries. There is a fine line between subtle amplification and tipping the face into distortion. Once that threshold is crossed, the face enters the “uncanny valley.” A neurobiological conflict state, when a face is sufficiently human‐like to recruit normal face‐processing and social‐cognition systems yet contains enough dynamic anomalies to violate expected priors [34]. In practice, aesthetic treatments that distort texture, contour, timing, or identity result in a face that is recognized as human, and perhaps even attractive, but not believable. The result is discomfort rather than delight.

## 11 Cultural Neuroscience and the Modulation of Beauty

While evolutionary pressures shape baseline sensitivity to facial cues such as symmetry, health, and vitality common to all individuals cultural experience shapes neural systems involved in perception, emotion, and social evaluation. Jack and colleagues demonstrated that facial expressions are not universal [35]. They identified measurable differences in facial movement patterns and expression between European and Asian subjects. Consistent with these findings, cross‐cultural perception studies by Cunningham and colleagues demonstrated meaningful variation in how expressive and affective cues are weighted across cultures [36]. In some populations, more subdued expressions may be associated with greater attractiveness whereas heightened expressivity in others is favored. This explains why emotional intensity and expressiveness in South American countries maybe perceived differently than in Eastern Asian Provinces. This provides support for the differences in cultures self‐ perception of emotions as well as differences in what each culture finds attractive. For clinicians, the practical importance underscores the need for cultural awareness and individualized consultation. It also helps explain why patient preference, and observer‐reported outcomes may vary by cultural context. It is not surprising that intradermal “meso‐Botox” or microtoxin strategies, have gained broader traction in some Eastern cultures than in Western ones [37]. Mild weakening of facial movement may not be consciously detectable, yet its subtle reduction of expressivity may in some settings be judged as more attractive or more desirable than it would be in others. Neuroaesthetic optimization takes into account the culturally specific ways faces are expressed, perceived, and socially valued.

## 12 Aesthetic Medicine, Mood, and Social Spread

Once these individual perceptual and emotional effects are recognized, it becomes easier to understand how aesthetic intervention can influence mood, confidence, body image, and quality of life. Changes in self‐perception, interpersonal response, and behavioral confidence become self‐reinforcing loops that then may even spread to those nearby. Fowler and Christakis, using longitudinal social‐network data from the Framingham Heart Study, reported that happiness clusters in social networks and may spread up to three degrees of separation [38]. While it is tempting to infer that aesthetic treatment “makes communities happier” a measured approach suggests through repeated social contact, micro‐affirmation, and small improvements in one's embodied confidence may create ripple effects in their environment. If you think about it, aesthetic treatments may have psychosocial consequences that extend beyond the single patient in the chair to influence the patient's immediate social world.

## 13 A Neuroaesthetic Feedback Framework

Taken together, these observations support a neuroaesthetic feedback model for facial aesthetic medicine:

1. *Intervention modifies facial input*. Cosmetic treatment alters structural, surface, and/or dynamic features of the face.
2. *Sensory–motor systems reprocess the face*. Configural processing, motion analysis, and embodied simulation generate an updated perception of expressivity and emotion for both the patient and observer.
3. *Meaning–knowledge systems interpret the change*. Identity, authenticity, age congruence, and cultural expectation shape how the change is understood by both the patient and observers.
4. *Observers respond socially*. Rapid first impression judgments and affective responses influence gaze, warmth, trust, attention, and behavior toward the patient.
5. *The patient receives feedback*. Self‐appraisal and social reinforcement or dissonance alter confidence, mood, and identity alignment.
6. *Effects consolidate over time*. These loops may strengthen positive or negative outcomes beyond the initial structural change and extend to those within immediate proximity.

This model integrates neurobiology and clarifies that an intervention's final effect is beyond the syringe tip or mirror immediately after treatment. Its real impact emerges through interaction among perceptual, affective, and social systems.

## 14 Clinical Translation: What Neuroaesthetic Thinking Changes in Practice

Neuroaesthetic thinking should change consultation, planning, technique, and outcome assessment. Consultation should expand beyond structural deficits to include expressive, social, and identity‐based concerns. Helpful questions include:

- What do people most often misread about your face?
- Are you more bothered by how you look at rest, in motion, or in photographs/ “selfies?”
- Do you want to look younger, less tired, less angry, softer, stronger, or simply more like yourself?
- Are you seeking improvement or transformation?
- Which facial expressions do you most want to preserve?

These questions help reveal whether the patient's concern is primarily structural, expressive, social, or self‐conceptual.

### 14.1 Treatment Planning

Neuroaesthetic planning emphasizes the endpoint is not wrinkle effacement or maximal projection, but a face that remains biologically plausible, age‐congruent, and socially legible. Step‐by‐step treatment algorithms are beyond which offers a practical framework for making small, strategic, high‐impact changes rather than obvious overcorrections. A detailed description on how to achieve these results is beyond the scope of this paper, but interested readers are referred to previously published descriptions [39]. Utilizing a subliminal difference strategy when treating aesthetic patients has been validated neatly within a neuroaesthetic model because it privileges subtle signal optimization over brute‐force transformation.

## 15 Outcomes in a Neuroaesthetic Era

Conventional endpoints used in aesthetic research remain essential, including wrinkle severity, structural correction, complication rates, and patient satisfaction. But these do not fully capture the psychosocial and neuroperceptual dimensions of outcome. A neuroaesthetic research agenda should also include patient and observer reported outcomes (PRO's, ORO's):

- Validated quality‐of‐life and appearance‐related measures such as FACE‐Q modules.
- Mood and affect measures
- Social confidence and functioning scales.
- Observer‐reported first‐impression paradigms using standardized photographs and video dynamic expression ratings
- Relationship and intimacy metrics

The goal is to measure dimensions patients care about and to build a framework that helps teach novice clinicians how to pursue reliably natural, socially meaningful, and identity‐congruent outcomes.

## 16 Conclusions

A neuroaesthetic framework negates narrow ideals, rigid algorithms, or facial homogenization. To emphasize cultural variability, neurobiologic plausibility and beauty that remains compatible with identity. If pursued responsibly, neuroaesthetics can help move aesthetic medicine away from a caricature of vanity and toward a more rigorous biopsychosocial science of attractiveness, well‐being, and human connection.

## Conflicts of Interest

Dr. Steven Dayan and Dr. Sabrina Fabi report professional relationships with companies in the aesthetic and dermatologic industries. These relationships are managed in accordance with institutional and journal policies. The authors declare that the content of this manuscript was developed independently and reflects their academic perspectives.

## Data Availability

The data that support the findings of this study are available from the corresponding author upon reasonable request. The data are not publicly available due to privacy and ethical restrictions related to participant confidentiality.
